# Novel *KLHL26* variant associated with a familial case of Ebstein’s anomaly and left ventricular noncompaction

**DOI:** 10.1002/mgg3.1152

**Published:** 2020-01-27

**Authors:** Sai Suma K. Samudrala, Lauren M. North, Karl D. Stamm, Michael G. Earing, Michele A. Frommelt, Richard Willes, Swarnendu Tripathi, Nikita R. Dsouza, Michael T. Zimmermann, Donna K. Mahnke, Huan Ling Liang, Michael Lund, Chien‐Wei Lin, Gabrielle C. Geddes, Michael E. Mitchell, Aoy Tomita‐Mitchell

**Affiliations:** ^1^ Department of Cell Biology, Neurobiology and Anatomy Medical College of Wisconsin Milwaukee WI USA; ^2^ Department of Otolaryngology and Communication Sciences Medical College of Wisconsin Milwaukee WI USA; ^3^ Department of Surgery Division of Cardiothoracic Surgery Medical College of Wisconsin Milwaukee WI USA; ^4^ Department of Pediatrics Children’s Hospital of Wisconsin Milwaukee WI USA; ^5^ Herma Heart Institute Children’s Hospital of Wisconsin Milwaukee WI USA; ^6^ Bioinformatics Research and Developmental Lab Genomic Sciences and Precision Medicine Center Medical College of Wisconsin Milwaukee WI USA; ^7^ Clinical and Translational Science Institute Medical College of Wisconsin Milwaukee WI USA; ^8^ Department of Obstetrics and Gynecology Medical College of Wisconsin Milwaukee WI USA; ^9^ Division of Biostatistics Medical College of Wisconsin Milwaukee WI USA; ^10^ Department of Biomedical Engineering Medical College of Wisconsin Milwaukee WI USA

**Keywords:** congenital heart defects, Ebstein's anomaly, Kelch‐like family member 26, left‐ventricular noncompaction, ubiquitin proteasome

## Abstract

**Background:**

Ebstein's anomaly (EA) is a rare congenital heart disease of the tricuspid valve and right ventricle. Patients with EA often manifest with left ventricular noncompaction (LVNC), a cardiomyopathy. Despite implication of cardiac sarcomere genes in some cases, very little is understood regarding the genetic etiology of EA/LVNC. Our study describes a multigenerational family with at least 10 of 17 members affected by EA/LVNC.

**Methods:**

We performed echocardiography on all family members and conducted exome sequencing of six individuals. After identifying candidate variants using two different bioinformatic strategies, we confirmed segregation with phenotype using Sanger sequencing. We investigated structural implications of candidate variants using protein prediction models.

**Results:**

Exome sequencing analysis of four affected and two unaffected members identified a novel, rare, and damaging coding variant in the Kelch‐like family member 26 (*KLHL26*) gene located on chromosome 19 at position 237 of the protein (GRCh37). This variant region was confirmed by Sanger sequencing in the remaining family members. *KLHL26* (c.709C > T p.R237C) segregates only with EA/LVNC‐affected individuals (FBAT *p* < .05). Investigating structural implications of the candidate variant using protein prediction models suggested that the KLHL26 variant disrupts electrostatic interactions when binding to part of the ubiquitin proteasome, specifically Cullin3 (CUL3), a component of E3 ubiquitin ligase.

**Conclusion:**

In this familial case of EA/LVNC, we have identified a candidate gene variant, *KLHL26* (p.R237C), which may have an important role in ubiquitin‐mediated protein degradation during cardiac development.

## Graphical abstract

Patient's Ebstein's anomaly (EA), a rare congenital heart disease of the tricuspid valve and right ventricle often manifest with left ventricular noncompaction (LVNC), a cardiomyopathy. Our study identifies a novel, rare, and damaging coding variant c.709C > T (p.R237C) in the Kelch‐like family member 26 (KLHL26) gene with 10 affected out of 17 affected members using Sanger sequencing. Through structural modeling, we show that the KLHL26 variant may affect protein binding to Cullin3 (CUL3), a component of E3 ubiquitin ligase in the ubiquitin‐mediated protein degradation.

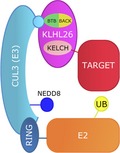

## 1 INTRODUCTION

Congenital heart defects (CHDs) are malformations of cardiac development and function, ranging from chamber to valve formation. CHDs account for a high burden of neonatal morbidity and mortality in the developed world (Attenhofer Jost, Connolly, Dearani, Edwards, & Danielson, 2007). Despite tremendous advances in genomic technologies, diagnostic variants cannot be identified for most patients. Therefore, additional approaches are required to elucidate the etiology of CHDs. Ebstein's anomaly (EA) is a rare CHD, comprising less than 1% of all cases with a prevalence of 1 in 200,000 live births (Attenhofer Jost et al., 2007). EA is characterized by the failure of posterior and septal tricuspid valve leaflets to delaminate, resulting in apical displacement of the valve, “atrialization” of the right ventricle above the valve, redundancy and fenestration of the anterior leaflet, and dilation of the atrioventricular junction (Attenhofer Jost et al., 2007). EA commonly manifests with left ventricular noncompaction (LVNC), a cardiomyopathy theorized to be due to hyper‐trabeculation or abnormal compaction of the ventricular trabeculations during normal development, causing sponge‐like myocardium (Pignatelli et al., 2014; Zhang, Chen, Qu, Chang, & Shou, 2013). In one study, 10 of 61 patients with EA (16.4%) also had LVNC (Pignatelli et al., 2014). Clinically, patients with EA can present at any age due to the varying degrees of anatomic and physiologic presentations of the disease. If degree of tricuspid valve insufficiency is mild, presentation can be delayed. On the other hand, presentation with severe tricuspid valve insufficiency can cause hydrops fetalis and death (Voges, Al‐Mallah, Scognamiglio, & Di Salvo, 2018). Signs and symptoms include cyanosis, dyspnea, right‐sided heart failure, hepatomegaly, cardiomegaly, and arrhythmias (Galea, Ellul, Schembri, Schembri‐Wismayer, & Calleja‐Agius, 2014). As such, management is individualized for each patient, in severe cases including surgical treatment such as tricuspid valve repair or replacement (Galea et al., 2014).

Both sporadic and familial EA cases have been associated with sarcomeric genes including myosin heavy chain 7 (*MYH7*) (Bettinelli et al., 2013; Postma et al., 2011) and filamin A (*FLNA*) (Mercer et al., 2017), transcription factor genes including *NKX2.5* (Benson et al., 1999; Gioli‐Pereira et al., 2010) and *GATA4* (Digilio et al., 2011), and channel genes such as sodium channel voltage gated type V (*SCN5A*) (Neu et al., 2010), all of which have a role in myocardial development (Sicko et al., 2016). Likewise, isolated and familial LVNC has been associated with various genes including tafazzin (*G4.*5) (Bione et al., 1996), Z‐band alternatively spliced PDZ motif‐containing protein (*ZASP*) (Vatta et al., 2003), transcription factor *TBX20* (Kodo et al., 2016), *MYH7* (Budde et al., 2007), myosin‐binding protein C (*MYBPC3*) (van Waning et al., 2018), titin (*TTN*) (van Waning et al., 2018), alpha‐dystrobrevin (*DTNA*) (Kenton et al., 2004), and FK‐binding protein‐12(*FKBP12*) (Kenton et al., 2004). Although genes such as *MYH7* (Vermeer et al., 2013), α‐tropomyosin (*TPM1*) (Kelle, Bentley, Rohena, Cabalka, & Olson, 2016; Nijak et al., 2018), and *NKX2.5* (Benson et al., 1999) have been implicated in both EA and LVNC, little is understood regarding the genetic etiology of this disease phenotype. Genetic heterogeneity, low recurrence, and variable clinical phenotypes indicate the interaction of multiple pathobiological mechanisms yet to be clearly defined in patients with EA and LVNC.

In this study we report a rare, novel variant of *KLHL26* in a family with autosomal dominant inheritance of EA/LVNC. This gene has not been previously associated with Mendelian disease. We describe multiple in vitro and computational approaches designed to characterize this variant and its potential physiologic functions.

## 2 METHODS

### 2.1 Ethics Statement

This study is in accordance with institutionally approved research (IRB) protocols by the Children's Hospital of Wisconsin (CHW) and conforms to the US Federal Policy for the Protection of Human Subjects. Subjects were consented through the IRB‐approved CHW ‐ CHD Tissue Bank (IRB #CHW 06/229, GC 300). This biorepository provided all DNA samples and cardiac tissue of patients and family members.

### 2.2 Family Pedigree

**Figure 1:**
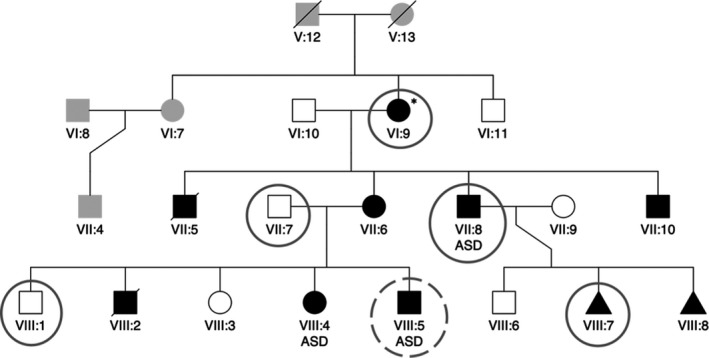
Pedigree of family with members affected by EA/LVNC. EA/LVNC‐affected members are denoted black. VII:5 was a stillborn male, VIII:2 was a premature birth, and VIII:7 was an IUFD; all were affected with cardiac malformations. VIII:8 was an IUFD at 8 weeks, also believed to be of cardiac origin. VI:9 does not fit the complete criteria for EA but is within the spectrum of the phenotype. The proband (VIII:5) is denoted by a dotted circle. Exome sequencing was performed on all encircled family members (VI:9, VII:7, VII:8, VIII:1, VIII:5, and VIII:7). EA/LVNC, Ebstein's anomaly with left ventricular noncompaction; IUFD, intrauterine fetal death

We identified this family after the proband (VIII:5) presented with severe EA/LVNC to the Children's Hospital of Wisconsin (CHW) (Figure 1). Historical information provided by the family and clinical information from medical records was used to create the pedigree. We performed all measurements of the four chambers, apical displacement of tricuspid valve, presence of LVNC, and degree of tricuspid insufficiency (Table 1 and Table S1). We recorded characteristics of EA according to diagnostic criteria including 1) tethering of tricuspid valve, 2) apical displacement of valve >8mm/m^2^ body surface area, and 3) combined area of right ventricle plus right atrium larger than combined area of right ventricle, left ventricle, and left atrium (ratio >1.0) (Attenhofer Jost et al., 2007). We extracted DNA (gDNA) from peripheral blood samples of family members and performed exome sequencing of VI:9, VII:7, VII:8, VIII:1, VIII:5, and VIII:7 (encircled blue in Figure 1). Methods of exome sequencing, variant filtering, and in silico modeling are described in the Data Supplement.

**Table 1:** Echocardiography results

Pedigree ID	Relationship	Age (yr)	LVNC?	TVd/BSA (mm/m^2^)	(RA + RV)/(RV + LV +LA)	EA?
VIII:5	Proband	Day 0	Yes	25.5	1.25	Yes
VII:7	Father	37	No	3.88	0.44	No
VII:6	Mother	37	Yes	14.94	0.98	Yes
VIII:4	Sister	2	Yes	20.69	1.05	Yes
VIII:1	Brother	12	No	5.23	0.73	No
VIII:3	Sister	10	No	6.58	0.63	No
VII:8	Maternal uncle	34	Yes	8.06	1.26	Yes
VI:9	Maternal grandmother^a^	—	No	4.31	—	No
VIII:6	Maternal first cousin	—	No	7.32	—	No
VI:11	Maternal great uncle	60	No	6.21	0.54	No

Abbreviations: BSA, body surface area; EA, Ebstein's anomaly; LA, left atrium; LV, left ventricle; LVNC, left ventricular noncompaction; RA, right atrium; RV, right ventricle; TVd, tricuspid valve displacement.

a Echocardiographic diagnostic criteria for Ebstein's anomaly include TVd/BSA of greater than 8 mm/m^2^ and/or combined right atrium RA and RV areas to be greater than areas of LV, RV, and LA combined (Attenhofer Jost et al., 2007). Maternal grandmother does not satisfy these criteria, but her echocardiogram did exhibit tricuspid anomaly and mild LVNC, which is within the spectrum of disease. Individual measurements can be found in Table S1.

## 3 RESULTS

### 3.1 Family Phenotyping

This family's CHDs pedigree (Figure 1) was revealed when prenatal ultrasound discovered severe EA/LVNC *in utero* for the proband (VIII:5), which was the mother's (VII:6) fifth pregnancy. This prompted our inquiry into the family's history of heart disease, which indicated the presence of cardiac anomalies in 10 of 17 family members. The mother's third living child (VIII:4) had previously undergone surgical repair for an atrial septal defect (ASD); it was also during this pregnancy that the mother herself (VII:6) was diagnosed with EA. Evaluation for CHDs confirmed an autosomal dominant inheritance pattern (Figure 1) for EA/LVNC. Echocardiography performed on her two other children (VIII:1 and VIII:3) indicated normal cardiac anatomy. The maternal grandmother (VI:9) had four pregnancies resulting in a stillborn male with cardiac defects (VII:5) and three living children with EA/LVNC (VII:6, VII:8, and VII:10).

The diagnostic criteria for EA/LVNC are defined by tricuspid valve displacement to body surface ratio greater than 8 mm/m^2^, and combined right atrial and right ventricular volume greater than combined right ventricular, left atrial, and left ventricular volume (Table 1) (Attenhofer Jost et al., 2007). Echocardiography and chart reviews revealed six family members with EA/LVNC (VI:9, VII:6, VII:8, VII:10, VIII:4, and VIII:5), and three with ASD (VII:8, VIII:4, and VIII:5). VIII:2 was a premature birth and VIII:7 was an intrauterine fetal death (IUFD) at 20 weeks gestational age; both demises were due to cardiac disease. VIII:8 was an IUFD at 8 weeks gestational age also theorized to be due to a cardiac defect. While the maternal grandmother (VI:9) did not qualify for EA according to the strict criteria, echocardiography indicated tricuspid valve anomaly and mild LVNC, which is within the spectrum of EA/LVNC. All affected individuals demonstrated some degree of tricuspid valve insufficiency (Table S1).

### 3.2 Exome Sequencing and Analysis

Exome sequencing of subjects VIII:5 and VII:8, followed by filtering with reference to a published differential mRNA expression pattern during murine cardiogenesis (Li et al., 2014), was employed to generate a gene candidate list. Although sequencing of several candidate genes did not reveal any variants segregating with EA/LVNC‐affected family members, additional exome sequencing of VI:9, VIII:1, and VIII:7 narrowed the list to six candidate genes, among which five were missense variants (Table S2). Two of the six candidate genes were also identified via a separate filtering method using Golden Helix VarSeq (described in the Data Supplement). Both filtering strategies resulted in two candidate variants—*RP1* (c.3532G > T p.D1178Y) and *KLHL26* (c.709C > T p.R237C). Sanger sequencing of the exon containing each candidate variant revealed that the *RP1* variant did not segregate with disease. However, the nonsynonymous variant on chromosome 19 (GRCh37) in the region of *KLHL26* (NM_001345981.1:c.709C > T p.R237C) segregated with EA/LVNC in all genotyped subjects (*n* = 14, eight affected and six unaffected; Figure 1a) (Family‐Based Association Test *p* < .05) and was fully penetrant. *KLHL26* (p.R237C), like other KLHL family members is involved in ubiquitin‐mediated protein degradation, but the specific functions of all family members have not been elucidated. KLHL26 is conserved across many species including chimpanzee, dog, rat, chicken, zebrafish, and frog.

### 3.3 Structural Modeling

**Figure 2:**
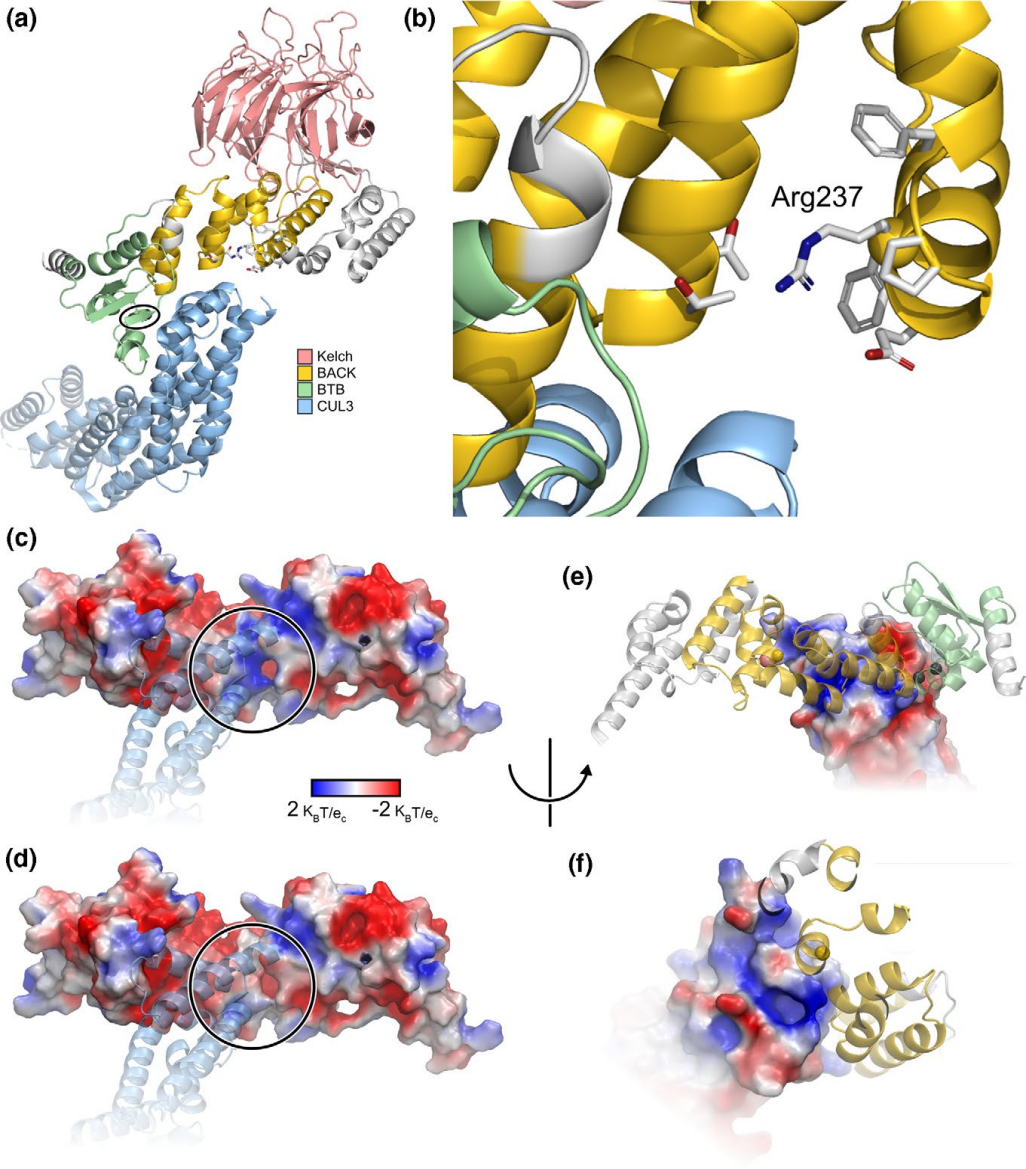
Structural Model of KLHL26‐CUL3 complex. (a) Our structural model is colored according to domain, depicting the predicted interaction with CUL3 mediated by the BACK and BTB domains. Residues outside of established domains by sequence comparison are colored white. Residues in the BTB domain that flank a site of additional sequence due to alternative splicing are circled. (b) Magnification of the BACK domain defines the location of R237 and its proximity to the CUL3 binding surface. (c). The electrostatic surface of KLHL26‐CUL3 interaction, wherein R237 is circled. (d) The analogous image of 2C for C237, wherein the electrostatic surface is more neutral and partially negative. (e) A 180° rotated view of the electrostatic surface of KLHL26‐CUL3 interaction; the gold circle marks the location of R237. (f) The positive patch on CUL3 is seen across from R237 (parts of KLHL26 that occlude the CUL3 surface are hidden). This patch is created by an HKH sequence whose sidechains fan out radially, contributing to a positive crescent‐shaped region, leaving the backbone oxygen atoms to make a negative surface patch

KLHL26 belongs to the Kelch‐like (KLHL) gene family and is a multidomain protein consisting of three types of domains (Dhanoa, Cogliati, Satish, Bruford, & Friedman, 2013) depicted in Figure 2a. The first is a homodimerization type domain known by two names: BTB (Broad‐Complex, Tramtrack and Bric a brac) and POZ (POxvirus and Zinc finger) (Stogios, Downs, Jauhal, Nandra, & Prive, 2005). The second domain is termed a BACK (BTB and C‐terminal Kelch) domain, named for its conserved presence in genes with BTB and Kelch domains. Third, KLHL26 has six Kelch repeats that form a single topologic fold (Stogios & Prive, 2004).

There are no published data describing the structure of KLHL26 protein. As an initial approach, we employed homology‐based modeling to assess KLHL26 structure. The BioPlex database (Huttlin et al., 2015) identifies with 99% probability that KLHL26 interacts with Cullin‐3 (CUL3), an important subunit of E3 ubiquitin ligase. Query of other genes in the KLHL gene family with prior data revealed that KLHL11 forms a complex with CUL3 (Canning et al., 2013). Therefore, we used this experimentally validated structure of KLHL11 to build a model of KLHL26 (Figure 2a,b). Although the R237C variant is located in the BACK domain of KLHL26, it is predicted to have little effect on stability (ΔΔGfold = 0.09 kcal/mol) based on FoldX (Parra et al., 2016). Amino acids are considered to be frustrated when favorable and unfavorable interactions are simultaneously experienced. Local frustration analysis identified R237 as a neutral amino acid, while C237 is minimally frustrated (energetically more stable), displaying a significant shift in frustration index, ΔF_R237C_ = F_C237_ − F_R237_ = 1.67 − (−0.41) = 2.08. Electrostatic profiling of the interface between proteins KLHL26 and CUL3 showed that whereas R237 forms a positive surface patch complementary to the negative CUL3 patch, the C237 variant forms a more neutral patch (Figure 2c‐f). This suggests that the C237 variant could alter KLHL26‐CUL3 binding affinity, perhaps decoupling the Cullin system in a fashion that disrupts the ubiquitin‐mediated pathway of protein degradation.

## 4 DISCUSSION

We have discovered that a rare, novel variant in *KLHL26* segregates with disease in a family with a highly penetrant form of EA/LVNC. In order to suggest an underlying pathogenic mechanism, we obtained a predicted molecular structure by comparing protein domains across the *KLHL26* gene family.

**Figure 3:**
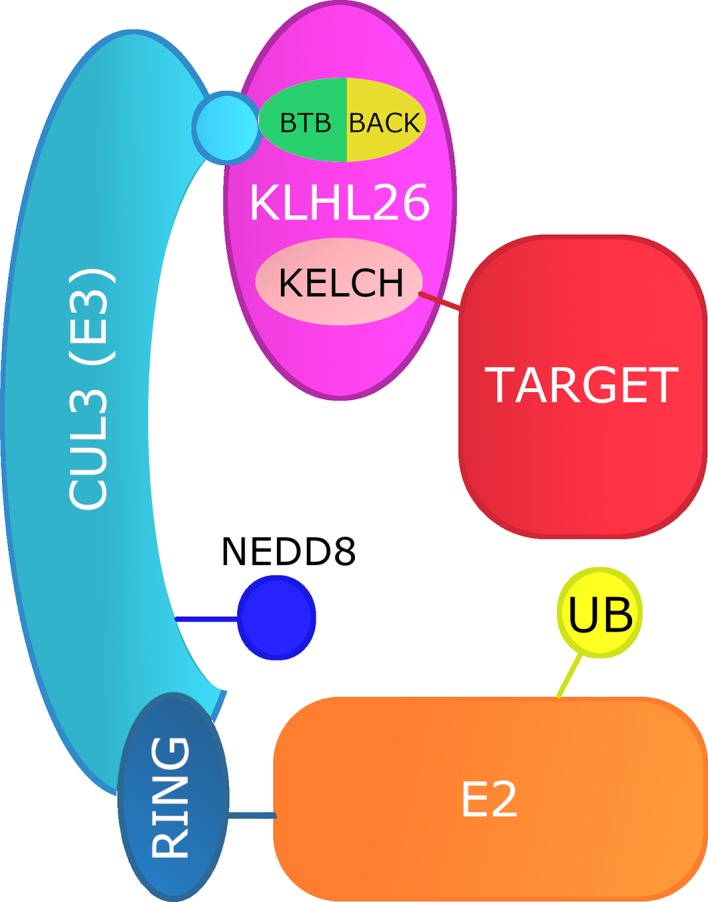
Proposed mechanism of KLHL26 interaction with CUL3. CUL3 is a core subunit of E3 ubiquitin ligase. Its N‐terminal domain binds to a receptor protein that confers substrate specificity – in this case KLHL26. The C‐ terminal domain binds a RING protein that recruits E2. NEDD8 brings substrate and E2 to the correct conformation for ubiquitination. In KLHL26, most likely the BTB domain interacts with CUL3 and the Kelch domain acts as the substrate‐recognition module

There is high probability that KLHL26 is a component of the ubiquitin‒proteasome system (UPS) (Hershko, Heller, Elias, & Ciechanover, 1983), wherein protein ubiquitylation occurs through a sequence of reactions catalyzed by E1, E2, and E3 enzymes, via its interaction with CUL3. The largest family of multisubunit E3 ubiquitin ligases is the cullin‐RING ligases (CRLs) (Petroski & Deshaies, 2005; Zimmerman, Schulman, & Zheng, 2010). KLHL family members are substrate adaptors for CUL3, which is part of the CRL3 subclass and is highly enriched in muscle tissues (Hori et al., 1999). The N‐terminal domain of CRLs binds to a substrate adaptor that recruits target proteins for ubiquitination, while the C‐terminal domain binds a RING protein that recruits E2. The target protein and E2 are brought into correct conformation through neddylation (NEDD8) of the C‐terminal domain. Both the BTB and BACK domains permit assembly with CUL3 (Canning et al., 2013). Other such domains commonly within the same protein as a BTB domain include C‐terminal Kelch, PHR, or zinc finger domains, with the Kelch β‐propeller domain being the most common for target recognition (Prag & Adams, 2003; Stogios et al., 2005). Thus, within KLHL26, the BTB and BACK domains likely interact with CUL3, while the Kelch domain recruit targets for ubiquitination (Figure 3). This model suggests that although R237C has little direct effect on KLHL26, it may significantly affect proteolytic function by altering protein:protein electrostatic interactions; as such, decoupling the Cullin system.

Considering the substantial physical intracellular forces exerted during the continual contraction of cardiac and skeletal muscle, it is not surprising that these cells have evolved mechanisms, including the UPS, to efficiently turn‐over sarcomeric proteins. Consequences of disrupting the CRL3‐KLHL pathway in striated muscle have been documented (Dhanoa et al., 2013; Papizan, Vidal, Bezprozvannaya, Bassel‐Duby, & Olson, 2018). KLHL40 (Bowlin, Embree, Garry, Garry, & Shi, 2013), KLHL41 (du Puy et al., 2012), KLHDC1 (Chin, Xu, Ching, & Jin, 2007; Sekine et al., 2012), and KLHDC2 (Chin et al., 2007; Sekine et al., 2012) are associated with myoblast differentiation. KLHL31 (Abou‐Elhamd, Cooper, & Munsterberg, 2009; Yu et al., 2008) and KLHL41 (Greenberg, Connelly, Daniels, & Horowits, 2008) have roles in muscle maturation. KLHL41 is also associated with myoblast proliferation (Paxton et al., 2011). KLHL19 (Miller et al., 2012), KLHL20 (Hara et al., 2004), ND1‐L (Sasagawa et al., 2002), and KLHL27 (I. F. Kim, Mohammadi, & Huang, 1999) either bind to actin or are associated with actin, playing critical roles in cytoskeletal arrangement. KLHL9 (Cirak et al., 2010), KBTBD13 (Sambuughin et al., 2010), KLHL40 (Ravenscroft et al., 2013), and KLHL41 (Gupta et al., 2013) have been implicated in distal and nemaline myopathy. CUL3 is enriched in muscle tissues, essential for myoblast differentiation, and conditional CM‐KO of CUL3 is neonatal lethal in mice (Blondelle, Shapiro, Domenighetti, & Lange, 2017; Papizan et al., 2018). CM deletion of NAE1, a regulatory subunit of neddylation, in mice CMs caused heart failure and perinatal lethality (Zou et al., 2018). In summary, substantial data exist implicating KLHL proteins in muscular function, in processes ranging from myoblast migration to cytoskeletal arrangement.

Evidence suggests that the EA/LVNC phenotype may result from sarcomeric disarray, as indicated by electron microscopic images revealing disrupted Z‐bands in the right atrium and atrialized part of the right ventricle of EA patients (Egorova, Penyaeva, & Bockeria, 2015). The authors suggested that the similarity between electron dense deposits observed in cardiomyocytes of EA patients and skeletal muscle myopathies occurring in patients harboring mutations in genes encoding α‐actin, nebulin, and titin might link EA pathogenicity to a sarcomeric protein, or perhaps to a transcription factor required to maintain the expression of sarcomeric genes. In this regard, RNA‐seq analysis performed on H1‐ESCs (Kim et al., 2015) showed that *KLHL26* is expressed during cardiomyocyte differentiation. As noted earlier, previously identified genetic risk factors for EA/LVNC are mainly comprised of sarcomeric proteins. We accordingly hypothesize that *KLHL26* (p.R237C) dysregulates the degradation of a sarcomeric protein, causing altered cardiomyocyte proliferation and differentiation, and ultimately results in presentation of the familial EA/LVNC phenotype.

In summary, while the specific function of KLHL26 in the UPS remains unknown, growing evidence that mutated KLHL proteins disrupt striated muscle homeostasis, combined with the high disease‐associated penetrance of this specific *KLHL26* variant, warrants investigation to validate the predictions suggested by the modeling described in Figure 3. In order to determine possible effects of disrupted UPS processing on the developing heart, we are modeling this familial EA/LVNC using induced pluripotent stem cells derived from the family depicted in Figure 1 to identify *KLHL26*‐dependent changes in cardiomyocyte proliferation and differentiation.

## DISCLOSURES

A. Tomita‐Mitchell and M. E. Mitchell are cofounders of TAI Diagnostics (Milwaukee, WI), a biotechnology company involved in transplant diagnostics, and members of its scientific advisory board.

## Data Availability

The data are not publicly available due to privacy or ethical restrictions.
