# Supplementary material for: Novel KLHL26 variant associated with a familial case of Ebstein’s anomaly and left ventricular noncompaction
Source: Mol Genet Genomic Med. 2020 Jan 27;8(4):e1152. doi: 10.1002/mgg3.1152 (PMC7196453; doi:10.1002/mgg3.1152)
Supplement: Supplementary file 1 [file MGG3-8-e1152-s001.docx]

**Supplemental Material**

Supplemental Methods

Exome sequencing

We exome sequenced on a HiSeq 2000 DNA sequencer (Illumina, San Diego, CA) at the Children’s Hospital of Wisconsin (CHW) Children’s Research Institute (Milwaukee, WI). Agilent SureSelectXT Human All Exon kit (v5; Agilent Technologies, Santa Clara, CA) was used to generate exome sequencing libraries. We then aligned samples with BWA(Li & Durbin, 2009) to genome reference GRCh37. Using two bioinformatic approaches, we assessed shared rare variants that segregated with EA/LVNC (Figure S1). In the first approach, we detected indel and sequence variations using the Broad GATK(McKenna et al., 2010), then annotated with GEMINI (Paila, Chapman, Kirchner, & Quinlan, 2013) and Ensembl VEP (v78) (McLaren et al., 2010).

Filtering Method 1

The list was then filtered for variants with the following characteristics: rare (mean allele frequency <1% NHLBI Exome Sequencing Project (ESP) EA and AA subgroups (URL: http://evs.gs.washington.edu/EVS/) [6500 samples, February, 2013]), and the 1000 Genomes Project (Wain et al., 2011) ASN, AMR, AFR, EUR and SAS subgroups), novel, damaging, non-intronic/untranslated region/coding-synonymous, GATK quality score >66 per subject, base pair locus conservation as indicated by Genomic Evolutionary Rate Profiling (GERP) >1, outside of a RepeatMasker domain, and without segmental duplication (Cooper et al., 2005). Additionally, the variant is predicted as damaging to protein function by PolyPhen2> 0.50 and/or by SIFT < 0.50 (Adzhubei et al., 2010; Ng & Henikoff, 2003). To remove technical artifacts, we made sure indel calls were no more than four base pairs in length, the site was called confidently among at least half of the cohort, and the indel was not found in more than 10% of sequenced subjects. We prioritized candidate genes by cardiac tissue expression and known association with cardiac or muscle development.

Filtering Method 2

In the second approach, variant call files (VCF) were analyzed using Golden Helix VarSeq Software v.1.2.1 (Bozeman, MT). In VarSeq, variants with read depth <15 and genotype quality score <20 were excluded. We considered only rare variants (mean allele frequency <0.2%; 1000 Genomes Project, Exome Aggregation Consortium (ExAC) (Lek et al., 2016), NHLBI ESP exomes, and Genome Aggregation Database (GnomAD) and those with a PHRED scaled Combined Annotation Dependent Depletion (CADD) (Kircher et al., 2014) score > 30 (top 0.1% of reference genome single nucleotide variants). Using the Count Alleles algorithm, we isolated variants that segregated with disease.

Sanger sequencing

From each candidate variant, we designed primers to target 100-150 base pairs up- and down- stream using NCBI human reference genome and Primer3 (v0.4.0) (Koressaar & Remm, 2007; Untergasser et al., 2012) primer design software. Double-stranded DNA from PCR amplification was purified with ExoSAP-IT (Affymetrix, Santa Clara, CA), sequenced by Retrogen, Inc (San Diego, CA), and analyzed using Sequencher v4.5 (Gene Codes Corporation, Ann Arbor, MI).

KLHL26 Structural Modelling

There are no experimental structures for the human KLHL26 protein. Based on available experimental structures for related proteins from the same family of Kelch motif proteins, we used RaptorX (Kallberg, Margaryan, Wang, Ma, & Xu, 2014) homology modeling and the Uniprot (The UniProt, 2017) canonical sequence, Q53HC5, to build the KLHL26 model. Homology modeling used multiple experimental structures as templates, primarily murine Keep1 (Kelch-like ECH-associated protein 1; 31% identical; PDB 5CGJ) and the human KLHL11-CUL3 complex (26% identical; PDB 4AP2). These templates were used to model the Kelch or BACK and POZ domains, respectively. Importantly, the KLHL gene family is known to have low sequence identity, yet conserved 3D structure, increasing our confidence in our model (Stogios, Downs, Jauhal, Nandra, & Prive, 2005). We used FoldX (v4.0) (Parra et al., 2016) to assess changes to the folding energy (ΔΔGfold), frustrometer (Parra et al., 2016) to assess the balance between favorable and unfavorable interactions between amino acids based on single residue level frustration, and gathered population allele frequency from GnomAD. Protein structure was visualized using PyMOL (v2.0.7) (Schrodinger, LLC, New York, NY). To test the feasibility for KLHL26 to interact with CUL3, we queried BioPlex (Huttlin et al., 2015): a database of high-throughput mass-spectrometry-based protein-protein interactions.

Supplemental Tables

**Table S1. Echocardiography measurements.**

| **Study ID** | **Sex** | **Degree of Relation** | **Body Surface Area (m^2^)** | **Tricuspid Valve Displacement**  **(mm)** | **Triscupid Insufficiency** | **Chamber Area (mm^2^)** | | | |
| --- | --- | --- | --- | --- | --- | --- | --- | --- | --- |
|  |  |  |  |  |  | **Right Atrium** | **Right Ventricle** | **Left Atrium** | **Left Ventricle** |
| VIII:5 | M | Proband | 0.2 | 5.1 | Severe | 6.17 | 1.39 | 1.88 | 2.8 |
| VII:7 | M | First | 2.01 | 7.8 | Physiologic | 10.02 | 14.86 | 7.7 | 34.52 |
| VII:6 | F | First | 1.68 | 25.1 | Moderate | 27.22 | 8.95 | 13.58 | 14.42 |
| VIII:4 | F | First | 0.58 | 12 | Mild | 9.96 | 5.23 | 3.16 | 6.11 |
| VIII:1 | M | First | 1.49 | 7.8 | Physiologic | 18.22 | 12.41 | 7.73 | 21.81 |
| VIII:3 | F | First | 1.2 | 7.9 | Physiologic | 11.85 | 7.16 | 7.56 | 15.67 |
| VII:8 | M | Second | 2.16 | 17.4 | Moderate | 40.17 | 11.44 | 16.71 | 12.84 |
| VI:9 | F | Third | 1.88 | 8.1 | Physiologic | 17.92 | - | 30.67 | - |
| VIII:6 | M | Third | 0.82 | 6 | Mild | 8.52 | - | 7.6 | - |
| VI:11 | M | Fourth | 1.95 | 12.1 | Physiologic | 13.88 | 9.87 | 14.71 | 19.19 |

**Table S2. Candidate gene list after filtering variants from exome sequencing of VI:9, VII:8, VIII:1, VIII:5, and VIII:7.**

| **Locus** | **Gene Symbol** | **Reference** | **Variant** | **Type** | **Amino Acid Change (Ref/Alt)** | **Impact** | **Poly Phen-2 Score** | **SIFT score** |
| --- | --- | --- | --- | --- | --- | --- | --- | --- |
| chr7 | *ACTB* | G | C | SNP | None | Splice region | None | None |
| chr8 | *RP1* | G | T | SNP | D/Y | Missense Variant | 0.982 | 0 |
| chr8 | *VCPIP1* | G | A | SNP | A/V | Missense Variant | 0.917 | 0.01 |
| chr8 | *PREX2* | C | T | SNP | S/L | Missense Variant | 0 | 0.1 |
| chr19 | *TMEM59L* | C | T | SNP | R/C | Missense Variant | 0.959 | 0.07 |
| chr19 | *KLHL26* | C | T | SNP | R/C | Missense Variant | None | None |

**Table S3: List of Primer Sequences for PCR.**

| **Gene Symbol** | **Primer Sequence** |
| --- | --- |
| *RP1* | F: TCAAGTTGATGCTCACAAGGC  R: AGAGCAACCTCCATCCAAAGAG |
| *KLHL26* | F: AGACCTGCCTCAACATCGG  R: AGCTCGGACGACTGCATG |

Supplemental References

Adzhubei, I. A., Schmidt, S., Peshkin, L., Ramensky, V. E., Gerasimova, A., Bork, P., . . . Sunyaev, S. R. (2010). A method and server for predicting damaging missense mutations. *Nat Methods, 7*(4), 248-249. doi:10.1038/nmeth0410-248

Cooper, G. M., Stone, E. A., Asimenos, G., Program, N. C. S., Green, E. D., Batzoglou, S., & Sidow, A. (2005). Distribution and intensity of constraint in mammalian genomic sequence. *Genome Res, 15*(7), 901-913. doi:10.1101/gr.3577405

Huttlin, E. L., Ting, L., Bruckner, R. J., Gebreab, F., Gygi, M. P., Szpyt, J., . . . Gygi, S. P. (2015). The BioPlex Network: A Systematic Exploration of the Human Interactome. *Cell, 162*(2), 425-440. doi:10.1016/j.cell.2015.06.043

Kallberg, M., Margaryan, G., Wang, S., Ma, J., & Xu, J. (2014). RaptorX server: a resource for template-based protein structure modeling. *Methods in molecular biology, 1137*, 17-27. doi:10.1007/978-1-4939-0366-5_2

Kircher, M., Witten, D. M., Jain, P., O'Roak, B. J., Cooper, G. M., & Shendure, J. (2014). A general framework for estimating the relative pathogenicity of human genetic variants. *Nat Genet, 46*(3), 310-315. doi:10.1038/ng.2892

Koressaar, T., & Remm, M. (2007). Enhancements and modifications of primer design program Primer3. *Bioinformatics, 23*(10), 1289-1291. doi:10.1093/bioinformatics/btm091

Lek, M., Karczewski, K. J., Minikel, E. V., Samocha, K. E., Banks, E., Fennell, T., . . . Exome Aggregation, C. (2016). Analysis of protein-coding genetic variation in 60,706 humans. *Nature, 536*(7616), 285-291. doi:10.1038/nature19057

Li, H., & Durbin, R. (2009). Fast and accurate short read alignment with Burrows-Wheeler transform. *Bioinformatics, 25*(14), 1754-1760. doi:10.1093/bioinformatics/btp324

McKenna, A., Hanna, M., Banks, E., Sivachenko, A., Cibulskis, K., Kernytsky, A., . . . DePristo, M. A. (2010). The Genome Analysis Toolkit: a MapReduce framework for analyzing next-generation DNA sequencing data. *Genome Res, 20*(9), 1297-1303. doi:10.1101/gr.107524.110

McLaren, W., Pritchard, B., Rios, D., Chen, Y., Flicek, P., & Cunningham, F. (2010). Deriving the consequences of genomic variants with the Ensembl API and SNP Effect Predictor. *Bioinformatics, 26*(16), 2069-2070. doi:10.1093/bioinformatics/btq330

Ng, P. C., & Henikoff, S. (2003). SIFT: Predicting amino acid changes that affect protein function. *Nucleic Acids Res, 31*(13), 3812-3814. Retrieved from <https://www.ncbi.nlm.nih.gov/pubmed/12824425>

Paila, U., Chapman, B. A., Kirchner, R., & Quinlan, A. R. (2013). GEMINI: integrative exploration of genetic variation and genome annotations. *PLoS Comput Biol, 9*(7), e1003153. doi:10.1371/journal.pcbi.1003153

Parra, R. G., Schafer, N. P., Radusky, L. G., Tsai, M. Y., Guzovsky, A. B., Wolynes, P. G., & Ferreiro, D. U. (2016). Protein Frustratometer 2: a tool to localize energetic frustration in protein molecules, now with electrostatics. *Nucleic Acids Res, 44*(W1), W356-360. doi:10.1093/nar/gkw304

Stogios, P. J., Downs, G. S., Jauhal, J. J., Nandra, S. K., & Prive, G. G. (2005). Sequence and structural analysis of BTB domain proteins. *Genome Biol, 6*(10), R82. doi:10.1186/gb-2005-6-10-r82

The UniProt, C. (2017). UniProt: the universal protein knowledgebase. *Nucleic acids research, 45*(D1), D158-D169. doi:10.1093/nar/gkw1099

Untergasser, A., Cutcutache, I., Koressaar, T., Ye, J., Faircloth, B. C., Remm, M., & Rozen, S. G. (2012). Primer3--new capabilities and interfaces. *Nucleic Acids Res, 40*(15), e115. doi:10.1093/nar/gks596

Wain, L. V., Verwoert, G. C., O'Reilly, P. F., Shi, G., Johnson, T., Johnson, A. D., . . . van Duijn, C. M. (2011). Genome-wide association study identifies six new loci influencing pulse pressure and mean arterial pressure. *Nat Genet, 43*(10), 1005-1011. doi:10.1038/ng.922
